# Effects of Methanol Fraction from Leaves of* Schinus terebinthifolius* Raddi on Nociception and Spinal-Cord Oxidative Biomarkers in Rats with Neuropathic Pain

**DOI:** 10.1155/2018/5783412

**Published:** 2018-05-03

**Authors:** Taína Scheid, Maira Silmara Moraes, Thiago Pereira Henriques, Ana Paula Konzen Riffel, Adriane Belló-Klein, Gilsane Lino Von Poser, Eduardo Miranda Ethur, Wania Aparecida Partata

**Affiliations:** ^1^Department of Physiology, Institute of Basic Health Sciences, University of Rio Grande do Sul (UFRGS), Porto Alegre, RS, Brazil; ^2^Institute of Pharmacy, University of Rio Grande do Sul (UFRGS), Porto Alegre, RS, Brazil; ^3^Department of Exact and Technological Sciences, UNIVATES, Lajeado, RS, Brazil

## Abstract

We determined the antioxidant potential of fractions obtained from leaves of* Schinus terebinthifolius*, a medicinal plant known in Brazil as aroeira, to select the fraction with the best yield and antioxidant performance. These qualities were found in the methanol fraction (MeF), which was administered intraperitoneally (20 mg/kg/day) for 3 and 10 days to rats with chronic constriction injury (CCI) of the sciatic nerve, a model of neuropathic pain. The MeF increased the mechanical and thermal thresholds that had been lowered by CCI. In parallel, the lumbosacral spinal cord showed an increase in superoxide dismutase but a decrease in glutathione peroxidase and glutathione-S-transferase activities in saline- and MeF-treated CCI rats. Catalase activity decreased only in saline-treated CCI rats for 10 days. Total thiols decreased in saline- and MeF-treated CCI rats. Ascorbic acid increased in these rats at day 3 but only in saline-treated CCI rats at day 10. No change was found in hydrogen peroxide and lipid hydroperoxide. Open-field and elevated plus-maze tests and blood parameters of liver function did not change. Thus, the MeF from leaves of* S. terebinthifolius* has an antinociceptive action with no toxic effects, and it affects oxidant biomarkers in the spinal cord of rats with CCI.

## 1. Introduction

Neuropathic pain, which arises as a direct consequence of a lesion or disease affecting the somatosensory system, affects 6.0–10% of the population and negatively impacts the quality of life of patients [1]. The pathophysiological mechanisms are not fully understood [2]. The lack of effective analgesic has impelled a continuing search to find novel molecules with beneficial effects in neuropathic pain. Natural products appear to be the most promising sources of new drugs [3], and the identification of bioactive compounds from plants that may be used to treat neuropathic pain has been a highly active area of pharmaceutical research.

Recently, Piccinelli et al. [4] demonstrated that the essential oil of* Schinus terebinthifolius *Raddi (Anacardiaceae), a plant native to South America and widely distributed in Brazil, popularly known as aroeira [5], induced antinociception in rats with spared nerve injury-induced neuropathic pain. In pain studies, each animal model has been created with a specific methodology, and the results tend to vary widely with slight changes in the methodology used to induce pain; therefore, it is essential that data from different pain models be reported and interpreted in the context of the specific pain model [6]. An answerable question is whether extracts from* S. terebinthifolius *have an antinociceptive effect in rats with chronic constriction injury (CCI) of the sciatic nerve. Rats with CCI are one of the most commonly employed animal models of neuropathic pain; CCI simulates the symptoms of chronic nerve compression that correspond to causalgia or complex regional pain syndrome in human patients [6].

Saponins, triterpenes, steroids [7], and phenolic compounds [8] have been found in extracts from* S. terebinthifolius*. Many of these compounds are scavengers of reactive oxygen species (ROS), which are formed in several circumstances including normal cellular metabolism, and participate in a number of functions, for instance, in pain modulation [9, 10]. Antioxidant agents have been tested to treat neuropathic pain [11, 12], but the effects of treatment with extracts from* S. terebinthifolius *on oxidative biomarkers in nervous tissue have not been investigated.

The present study was designed to investigate the antinociceptive effect of a fraction rich in antioxidant compounds obtained from* S. terebinthifolius* in rats with CCI-induced neuropathic pain and also to explore the effect of this fraction on prooxidant and antioxidant markers in the lumbosacral spinal cord. Since the yield of the methanol fraction (MeF) was highest and this fraction showed good antioxidant activity, this fraction was chosen to be intraperitoneally administered (20 mg/kg/day) for 3 and 10 days in rats with CCI. Then, the activities of superoxide dismutase (SOD), catalase (CAT), glutathione peroxidase (GPx), and glutathione-S-transferase (GST) and the total contents of thiols and ascorbic acid were assessed as antioxidant status markers in the lumbosacral spinal cord of rats with CCI. We also assessed the levels of lipid hydroperoxides and hydrogen peroxide (H_2_O_2_) in this tissue, as prooxidant status markers.

Our study also evaluated the locomotor and anxiety-like behaviors of the rats, using open-field (OF) and elevated plus-maze (EPM) tests, respectively; the blood parameters of gamma-glutamyltransferase (GGT), aspartate aminotransferase (AST), alanine aminotransferase (ALT), and bilirubin; and the body weight in naive rats 10 days after treatment, to reveal possible side effects of the treatment.

## 2. Materials and Methods

### 2.1. Plant Material and Preparation of Fractions

After authorization by the Conselho de Gestão do Patrimônio Genético (CGEN – 010738/2013-4), leaves of* S. terebinthifolius* were collected at Lajeado, Rio Grande do Sul, Brazil, and authenticated through the Department of Botany, Federal University of the Rio Grande do Sul, where the voucher specimen (number 166738) was deposited. The fractions were obtained by successive extractions of dried and powdered leaves, starting with n-hexane (He) and followed by dichloromethane (Dc), ethyl acetate (Et), and methanol (Me) by static maceration (0.5 L of solvent, daily exchanges for 12 days, for each solvent). The extractive solutions were evaporated to dryness under vacuum at low temperature (42 ± 2°C) and stored at 8°C. The yields of the fractions were HeF (3.84%), DcF (1.49%), EtF (1.63%), and MeF (5.30%).

### 2.2. Antioxidant Properties of* Schinus terebinthifolius* Fractions

*2,2-Diphenyl-1-picrylhydrazyl (DPPH) Radical Scavenging Activity. *The free radical scavenging activity of the fractions and antioxidant standards ascorbic acid and butylated hydroxytoluene (BHT) in different concentrations (25–500 *μ*g/mL) were measured [13]. All experiments were repeated three times independently. The percentage inhibition of DPPH free-radical scavenging activity was calculated using the following equation: DPPH inhibition% = [(*A*_0_ − *A*_1_)/*A*_0_ × 100], where *A*_0_ is the absorbance of the control reaction (3 mL of methanol and 1 mL of DPPH solution, without a sample of extracts) and *A*_1_ is the absorbance in the presence of the fractions or standards. The % inhibition data were then plotted versus the concentration and graphed, and the IC_50_ (half-maximal inhibitory concentration) value was calculated by linear regression analysis.

*Hydroxyl Radical Scavenging Activity. *This assay quantified hydroxyl radicals indirectly through the 2-deoxy-D-ribose degradation product, malondialdehyde, as described by Chobot [14], using fractions and BHT in different concentrations (25–250** *μ***g/mL). Assays were performed in triplicate and the results were expressed in IC_50_ values.

*Total Reactive Antioxidant Potential (TRAP). *The TRAP was determined by measuring the chemiluminescence (CL) intensity of luminol induced by ABAP, as described by Dresch et al. [15]. The CL was measured in a liquid scintillation counter (LKB Rack Beta Liquid Scintillation Spectrometer-*1215*, Sweden) using fractions at concentrations of 2.5, 5.0, 10.0, and 25.0 *μ*g/mL. The Trolox equivalents of the sample were calculated using a standard curve of Trolox.

*Phytochemical Screening. *Qualitative phytochemical analysis of* Schinus terebinthifolius* fractions was carried out by TLC (Silicagel, 60 F_254_) to evaluate the presence of alkaloids, anthracene derivatives, triterpenes/steroids, phenolic compounds in general, coumarins, flavonoids, and saponins as described by Wagner and Bladt [16].

*Determination of Total Phenolic Content. *The total phenolic content was determined as described by Siatka and Kašparová [13]. Results were expressed as milligram-equivalents of gallic acid per g of extract (EGA mg/g). All analyses were carried out in triplicate.

### 2.3. Animals and Treatment

Animal procedures were approved by the Ethics Committee of the Federal University of Rio Grande do Sul (# 19388). Male Wistar rats (230–290 g) were randomly and blindly divided into three experimental groups (naive, Sham, and CCI), and each was further divided into two subgroups (*n* = 6/subgroup), which received MeF at a dose of 20 mg/kg/day or 0.9% saline solution, intraperitoneally, for 3 and 10 d. The choice of dose was guided by previous studies that also employed alcoholic extracts of* S. terebinthifolius* and showed anti-inflammatory effects [17] and absence of genotoxic and mutagenic effect [18]. The time of study (3 and 10 days) was chosen because our previous studies showed that the spinal-cord oxidative biomarkers changed in rats with CCI-induced neuropathic pain [12]. Since the literature contains study showing that the extract of* S. terebinthifolius* may be used with both routes of administration (oral and intraperitoneal) [19], intraperitoneal administration was chosen because the basic goal of this drug delivery is to increase the local drug concentration and the duration of drug exposure while decreasing systemic drug toxicity [20]. The administration started on the day of surgery (after recovery from anesthesia) and was performed daily at 17:00 h by the same researcher.

### 2.4. Induction of Chronic Constriction Injury (CCI)

After anesthesia (90 mg/Kg ketamine and 10 mg/Kg xylazine), the right common sciatic nerve was exposed proximal to its trifurcation via a mid-thigh incision, and four ligatures (4.0 Shalon Chromic Catgut) were tied loosely around it as described by Bennett and Xie [21], with slight modifications [12]. After nerve ligation, the muscle and skin layer were immediately sutured with thread and a topical antibiotic was applied. To expose the sciatic nerve in Sham rats, all surgical procedures involved in the CCI were used except the ligature.

### 2.5. Thermal and Mechanical Thresholds

Rats were subjected to sensitivity assessments before the surgical procedure (day 0) and at 3, 5, 7, and 10 days after surgery. Thermal hyperalgesia was measured by placing the rats on a hot plate maintained at 50°C (±2°C). Withdrawal latency was considered as when the animal jumped or licked a hind paw, independently of the side. A cutoff time of 30 s was employed to prevent tissue injury.

To measure mechanical sensitivity, the responses of the injured hindpaw to electronic von Frey apparatus (Insight, Brazil) were measured. For this, increasing pressure was applied against the plantar surface. A positive response was indicated by an abrupt withdrawal of the paw, and the intensity of the pressure was automatically recorded (in grams). A single trial consisted of five applications of the plastic tip, once every 5–10 s. The mean of five readings was taken as the threshold for a particular timing trial.

Behavioral assessments were conducted at the same time of day (08:00 h) and by the same researcher.

### 2.6. Locomotor and Anxiety-Like Behaviors

Naive rats were divided into two groups (*n* = 12/group) that received saline or MeF (20 mg/kg/d, intraperitoneally). The tests were performed in the morning, but on separate days to avoid stress to the animals. The OF were performed after 9 days of treatment (day 10 after CCI) and the EPM were conducted after 10 days of injections (day 11 after CCI). The EPM was assessed as described by de Souza et al. [22]. For the OF, an apparatus that consists of a brightly illuminated circular arena (90 cm diameter, 50 cm-high walls) with the floor marked in 12 sectors by concentric circles was used.

### 2.7. Preparation of Tissue Samples

The body weight was evaluated in naive rats that received saline and MeF (20 mg/kg/d) for 10 days. The weight of animals was recorded before the start of the injections (day 0) and at the end of the treatment period (day 11).

All rats were killed by decapitation and blood and lumbosacral spinal cord were promptly collected. The blood was centrifuged (Sorvall RC 5B, Rotor SM 24) for 20 min at 1000 ×g and the plasma was used to determine GGT, AST, ALT, and bilirubin (Labtest). Commercially available kits (LABTEST) were used for these assays.

The spinal cord was immediately divided into two parts. One part was cooled in liquid nitrogen and processed to determine H_2_O_2_. Another part was homogenized in 1.15% KCl diluted 1 : 5 (w/v) containing 1 mmol/L phenylmethylsulfonyl fluoride. The homogenates were centrifuged at 800 ×g for 20 min at 4°C to remove the nuclei and cell debris. The supernatant was frozen at −80°C and used for assays of the antioxidant parameters.

### 2.8. Determination of Antioxidant Enzyme Activities

The activity of SOD was measured based on its action to neutralize the superoxide radicals to prevent oxidation of adrenalin to adrenochrome, a colorful byproduct that can be measured at 480 nm. The reaction medium contained glycine buffer (50 mM, pH 11.3) and adrenalin (60 mM, pH 2.0) and the results were expressed as units per milligram of protein [23].

The CAT activity was determined at 240 nm in a reaction medium containing phosphate buffer (0.1 M, pH 7.4) and H_2_O_2_ (0.88 M), and the results were expressed as pmol of H_2_O_2_ reduced per minute per milligram of protein [24].

The GPx activity was measured at 340 nm and the reaction medium contained phosphate buffer (140 mM, pH = 7.5), EDTA (1 mM), NADPH (0.24 mM), sodium azide (1 mM), GSH (5 mM), glutathione reductase (0.25 U/mL), and tertiary butyl hydroperoxide (0.5 mM) [25]. Results were expressed as nmoles of peroxide/reduced hydroperoxide per minute per milligram of protein.

GST activity, expressed as nanomoles per milligram of protein, was measured by the rate of formation of dinitrophenyl-S-glutathione at 340 nm [26].

### 2.9. Determination of Ascorbic Acid and Total Thiol Levels

Ascorbic acid (AA) concentration was determined according to method described by Roe and Kuether [27]. The assay mixture contained 0.3 mL homogenate treated with charcoal and filtered, 0.01 mL 10% thiourea, and 0.075 mL 2% 2,4-Dinitrophenylhydrazine and was incubated at 37°C for 3 h. Following this, color was produced by adding 0.375 mL 85% sulfuric acid and the absorbance was read at 540 nm. Standard curve was prepared by using different concentration of AA and slope was used to express amount of AA as micromole of AA per milligram of protein.

The total thiol content was determined as described by Aksenov and Markesbery [28]. The reaction medium contained phosphate/EDTA buffer (pH = 7.5) and 5,5′-dithiobis (2-nitrobenzoic) acid (DTNB, 10 mM). After 30 minutes of incubation, the absorbances were read at 412 nm. Results were expressed as micromoles of TNB per milligram of protein.

### 2.10. Determination of H_2_O_2_ and Lipid Hydroperoxides Levels

The assay was based on horseradish peroxidase- (HRPO-) mediated oxidation of phenol red by H_2_O_2_, leading to the formation of a compound that is absorbed at 610 nm. Sections of fresh tissue from the lumbosacral spinal cord were incubated for 30 min at 37°C in 10 mmol/L phosphate buffer (140 mmol/L NaCl and 5 mmol/L dextrose). The supernatants were transferred to tubes with 0.28 mmol/L phenol red and 8.5 U/mL HRPO. After 5 min incubation, 1 mol/L NaOH was added, and the solution was read at 610 nm. The results were expressed in *μ*moles H_2_O_2_ per mg tissue [29].

The lipid hydroperoxides were measured by oxidation of Fe^2+^ by LOOH in an acid medium containing xylenol orange dye, which forms a complex with Fe^3+^, as described by Jiang et al. [30]. Results are expressed in nmol per g tissue.

### 2.11. Protein Measurement

Protein was measured by the method of Lowry et al. [31], using bovine serum albumin as the standard.

### 2.12. Statistical Analysis

Data were analyzed by two independent researchers; one was blinded to treatment. Data for antioxidant assays of the extract were analyzed by one-way ANOVA followed by Tukey's post hoc test. Pearson's correlation coefficient (*r*) was calculated between total phenolic content and the methods used to assess the antioxidant activity. Data for von Frey and hot-plate tests were analyzed by repeated-measures ANOVA followed by Tukey's test. The results for EPM, plasma parameters, and body weight were analyzed by unpaired Student's *t*-test, while results of the OF were assessed by Mann–Whitney *U* test. Data for spinal-cord oxidative biomarkers were compared by three-way ANOVA followed by Holm-Sidak post hoc test. Differences were considered statistically significant when *P* was <0.05. All statistical analyses were carried out with the software Origin 8.

## 3. Results

### 3.1. Antioxidant Properties of* Schinus terebinthifolius* Fractions

In the hydroxyl scavenging assay, the HeF and EtF showed IC_50_ values similar to the antioxidant standard. The DcF and MeF showed higher values (*P* < 0.001) (Table 1). In the DPPH assay, the MeF and EtF showed excellent scavenging activities, superior to BHT and similar to ascorbic acid. The HeF and DcF, in turn, showed weaker activities, with IC_50_ values at least 80 times higher than the other fractions (*P* < 0.001). In the TRAP assay, the greatest potential was found in the MeF, followed by the EtF, DcF, and HeF (*P* < 0.001). The highest content of total phenols was found in the MeF, while the lowest occurred in the DcF and HeF (*P* < 0.001). A high linear correlation (*r* > 0.98; *P* < 0.05) was observed between TRAP values and phenolic content. No linear correlation was found in other antioxidant tests.

### 3.2. Phytochemical Screening of* Schinus terebinthifolius* Fractions

Anthraquinones and triterpenes/steroids were present in all fractions. Flavonoids and saponins were present only in the EtF and MeF. Coumarins were found only in the MeF (Table 1).

### 3.3. Behavioral Assessment

**Figure 1:**
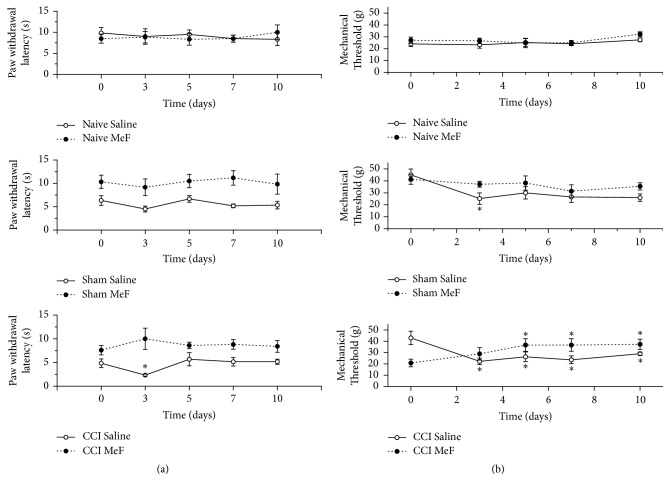
Assessment of behavioral hypersensitivity after chronic constriction injury (CCI) of the sciatic nerve in rats treated with methanol fraction (MeF) (20 mg/kg/day, i.p.) from leaves of* Schinus terebinthifolius *for 10 days. (a) Latency time against heat stimuli; (b) mechanical hypersensitivity. Data represent the means ± SEM (*n* = 6 for each group). *∗* indicates a significant difference compared to pre-nerve lesion values (repeated-measures ANOVA followed by Tukey post hoc test, *P* < 0.05).

After CCI, no rat displayed gross deficits in motor behavior that might have influenced the assessment of thermal and mechanical sensitivities. No significant change was found in the naive group throughout the experimental period (Figure 1).

At day 3 after surgery, the paw-withdrawal latency decreased (48.2%) in the saline-treated CCI rats, while no significant reduction was found in the MeF-treated CCI rats, compared to the pre-nerve-lesion level (Figure 1(a)). At days 5, 7, and 10, no significant change was found in the saline- and MeF-treated CCI rats compared to before nerve lesion. Despite baseline differing between saline- and MeF-treated sham rats, sham groups showed no significant changes in paw-withdrawal latency throughout the experimental periods.

The mechanical threshold in the saline-treated CCI rats decreased around 51% at day 3 compared to pre-nerve-lesion levels. The same response was found at days 5, 7, and 10. However, the MeF-treated CCI rats showed no decrease in the mechanical threshold (Figure 1(b)). At day 3 after surgery, the mechanical threshold of the MeF-treated CCI rats was similar to the pre-nerve-lesion threshold. However, a significant increase occurred at later time points: at 5, 7, and 10 days after surgery, the mechanical threshold was increased (76%) in the MeF-treated CCI rats compared to before nerve lesion.

Similar to the saline-treated CCI rats, the mechanical threshold in the saline-treated Sham rats also showed a reduction (56%), although only at day 3 compared to pre-procedure levels. No significant difference between the pre- and post-procedure levels was found at days 5, 7, and 10. The MeF-treated Sham rats showed no decrease in the mechanical threshold 3 days after surgery (Figure 1(b)).

No significant difference was found in the EPM and OF results for the naive rats treated with saline or MeF for 10 days (Table 2).

### 3.4. Effects on Biochemical Parameters of the Spinal Cord

**Figure 2:**
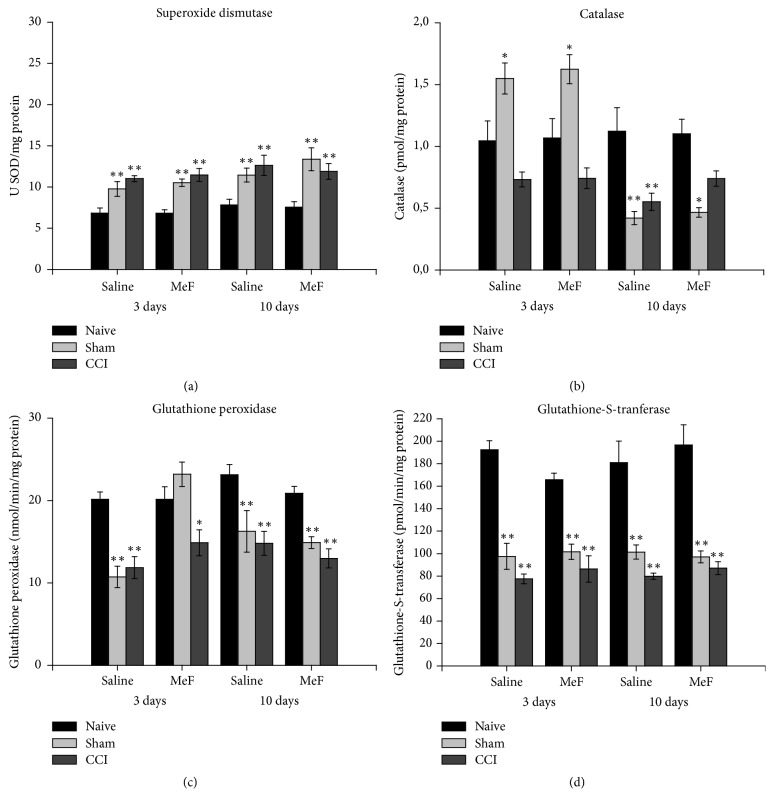
Activities of (a) superoxide dismutase, (b) catalase, (c) glutathione peroxidase, and (d) glutathione-S-tranferase in spinal cord of rats treated with methanol fraction (MeF) (20 mg/kg/d) or saline for 3 and 10 days. Data represent mean ± SEM (*n* = 6/group). *∗* indicates a significant difference compared to naive and* Sham* or naive and CCI at same treatment and experimental period. *∗∗* indicates a significant difference compared to naive at same treatment and experimental period (*P* < 0.05; three-way ANOVA followed by Holm-Sidak post hoc test).

At days 3 and 10 after CCI, the spinal-cord SOD activity increased by approximately 60% in the saline- and MeF-treated CCI rats. The saline- and MeF-treated Sham rats also showed significant increases in this parameter at days 3 and 10, compared to the naive group (Figure 2(a)). While the increase was 42 and 54% in the saline- and MeF-treated Sham rats, respectively, at day 3, the corresponding percentages were 46 and 78% at day 10.

At day 3, no significant change was found in spinal-cord CAT activity in the saline- and MeF-treated CCI rats. While this treatment did not produce a significant change in the MeF-treated CCI rats at day 10, the saline-treated CCI rats showed a decrease (46%) at this time point (Figure 2(b)). In Sham rats, CAT activity increased 48 and 51% in the spinal cord of the rats that received saline and MeF for 3 days, respectively. However, CAT activity decreased by around 60% in the saline- and MeF-treated Sham rats at day 10.

At days 3 and 10 after CCI, GPx activity was significantly reduced in the spinal cord of the saline- and MeF-treated CCI rats (Figure 2(c)). In Sham rats, while GPx activity decreased in animals that received saline for 3 days, the activity of this enzyme showed no significant change in the MeF-treated rats at this time point. At day 10, GPx activity decreased in both the saline- and MeF-treated Sham rats.

The GST activity decreased around 45% in the spinal cord from CCI rats that received MeF and saline for 3 and 10 days, compared to naive rats. In the Sham rats the activity of this enzyme also decreased. The reduction was approximately 50% in the saline- and MeF-treated Sham rats after 3 and 10 days (Figure 2(d)).

**Figure 3:**
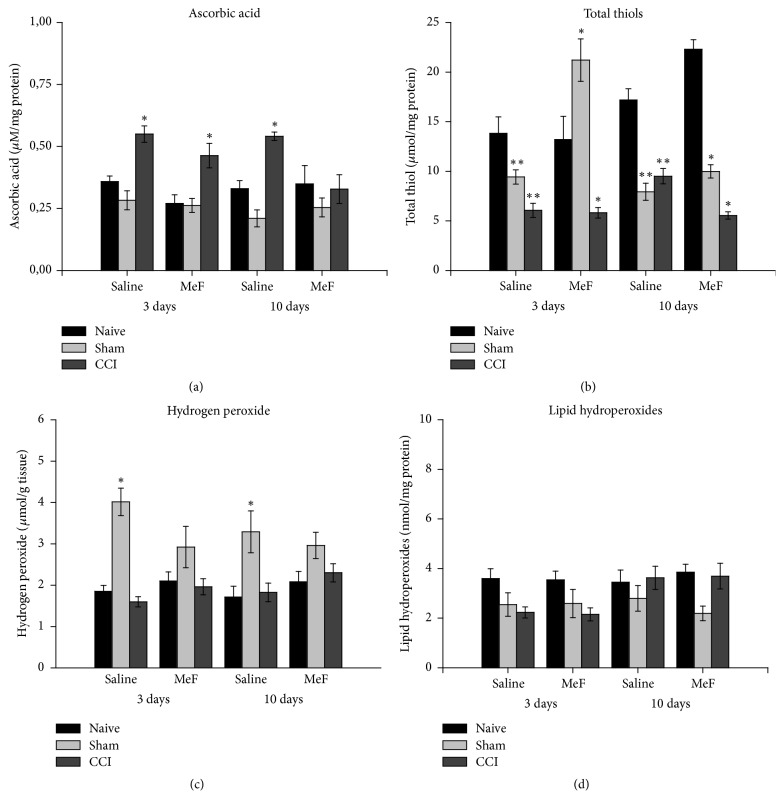
Ascorbic acid (a), total thiols (b), hydrogen peroxide (c), and lipid hydroperoxides (d) levels in spinal cord of rats treated with methanol fraction (MeF) (20 mg/kg/d) or saline for 3 and 10 days. Data represent mean ± SEM (*n* = 6/group). *∗* indicates a significant difference compared to naive and* Sham* or naive and CCI at same treatment and experimental period. *∗∗* indicates a significant difference compared to naive at same treatment and experimental period (*P* < 0.05; three-way ANOVA followed by Holm-Sidak post hoc test).

The ascorbic-acid content increased more than 50% in the spinal cord of CCI rats that received MeF and saline for 3 days, compared to naive rats. At day 10, only the saline-treated CCI rats showed an increase in ascorbic-acid levels. In the spinal cord of the MeF-treated CCI rats, the ascorbic-acid level was similar to that found in naive rats (Figure 3(a)). No significant change was found in Sham rats.

At days 3 and 10 after CCI, the total thiol was reduced in the spinal cord of the saline- and MeF-treated rats. At day 3, the decrease was around 43% in the spinal cord of these rats. At day 10, the decrease was 50% in the spinal cord of the saline-treated CCI rats, but approximately 28% in the spinal cord of the MeF-treated CCI rats (Figure 3(b)). In Sham rats, the total thiol content decreased in the spinal cord of the rats that received saline for 3 days, but increased (57%) in the MeF-treated rats. At day 10, there was a reduction of 43% in the spinal-cord total thiol content in the saline- and MeF-treated Sham rats.

No significant change was found in H_2_O_2_ levels in the spinal cord from the saline- and MeF-treated CCI rats after 3 and 10 days (Figure 3(c)). The MeF administration did not change the H_2_O_2_ level in the spinal cord from Sham rats that received treatment for 3 and 10 days. However, this prooxidant marker increased 122 and 88% in rats that received saline for 3 and 10 days, respectively.

Despite high variation, there was no significant difference in spinal-cord lipid hydroperoxide levels in CCI and Sham rats that received MeF and saline for 3 and 10 days (Figure 3(d)).

### 3.5. Effect on Blood Parameters and Body Weight

GGT, AST, ALT, and bilirubin levels did not change significantly with MeF administration (Table 3). Similarly, the MeF did not induce changes in body weight (initial: 234 ± 7 g; final: 256 ± 5 g) compared to the saline group (initial: 245 ± 11 g; final: 287 ± 14 g).

## 4. Discussion

The present study showed that extracts prepared from* S. terebinthifolius* leaves, particularly the MeF, have high antioxidant potential. The occurrence of an antioxidant potential in extracts from* S. terebinthifolius *is in line with the previous study by El-Massry et al. [8].

Our study showed for the first time that administration of the MeF induces attenuation of CCI-induced neuropathic pain. Since antioxidants are candidates for the treatment of neuropathic pain [11, 12], it is possible that the increase in the thermal and mechanical sensitivities of the MeF-treated CCI rats is related to the antioxidant potential of the MeF. However, the screening also indicated the presence of triterpenes/steroids and saponins in this fraction. These kinds of compounds also show an ameliorative effect on neuropathic pain [32], and therefore may also be contributing to the antinociceptive effect of the MeF. Another contributor to antinociception may be the anti-inflammatory effect of* S. terebinthifolius*. Some compounds of extracts from this plant appear to have anti-inflammatory activity [33]. Câmara et al. [34] demonstrated that reduction of the inflammatory process contributes to antinociception in rats with CCI. Thus, more tests are necessary to better characterize the chemical compounds of the MeF that may be contributing to antinociceptive effect of this fraction in rats with CCI.

The reduction in the mechanical threshold in the Sham rats may be due to the procedures involving manipulation of deep tissues, such as muscles and adjacent connective tissue, which induce pain [10]. Since MeF administration induced an antinociceptive effect in these animals, this result also reinforces the antinociceptive effect of the MeF in pain conditions. The lack of significant changes in the paw-withdrawal latency in the saline-treated CCI rats 5, 7, and 10 days after the surgery suggests a reversion of the thermal hyperalgesia, but we do not believe that a total reversion of symptoms occurs within 5 days. This may indicate a limitation of the test used in our study.

Our study also focused on ROS because of their emerging role in the pain mechanism [9–12]. For the first time, we demonstrated that the administration of an extract from* S. terebinthifolius* induced changes in spinal-cord prooxidant and antioxidant markers. Interestingly, the changes were not exclusive to the MeF-treated CCI rats, but some of them were also present in the saline-treated CCI rats and the saline- and MeF-treated Sham rats. These results suggest that the changes may be more related to the peripheral lesion than to the treatment. However, while the ascorbic-acid content increased in the saline-treated CCI rats after 10 days, this content was reduced in the MeF-treated CCI rats at this time point. This decrease suggests that the treatment induced a modulation in ascorbic acid. The increase in the ascorbic-acid content 3 days after CCI may indicate that the modulation of ascorbic acid occurred later. Ascorbic acid is involved in the first line of antioxidant defense in the brain, protecting the lipid membranes and proteins from oxidative damage [35]. Extracellular ascorbic-acid concentrations are increased in response to brain activity [35]. Glutamate and other neurotransmitters are increased in neuropathic pain [1, 2]. Thus, the increase in ascorbic-acid levels may be related to high synaptic activity in the spinal cord of rats with CCI. The reduction may be related to antioxidant molecules present in the MeF. Ji et al. [36] suggested that scavenging ROS appears to present an opportunity to normalize brain functions associated with pain. Since the MeF treatment did not decrease ascorbic-acid levels after 3 days, this result suggests that the modulation of ascorbic acid by MeF occurs later. Differences in the time of modulation may be related to the dose used in our study. Further studies with high doses of the MeF may help to clarify this suggestion.

When ascorbic acid carries out its antioxidant activity, it becomes oxidized. However, the reduction of its oxidized form is an enzymatic reaction, which may depend on glutathione [35], the most abundant thiol in mammals. Since ascorbic acid was increased in the CCI rats, the use of glutathione to reduce the oxidized form of ascorbic acid may have contributed to the decrease in the total thiols in the spinal cord of the CCI rats. Glutathione is a cofactor for GPx and GST [37]. The reduction in glutathione may explain the decrease in GPx and GST activities. In the Sham rats, the reduction in total thiols may be indicating the effects of these molecules. Total thiols constitute a group of molecules that act as cofactors in some enzymatic systems, and they can directly neutralize radicals [37]. The reduction in total thiols may be due to the important role of the antioxidants in muscle regeneration. Kozakowska et al. [38] demonstrated that oxidative stress is an important modulator of skeletal muscle regeneration after injury. The glutathione reduction may explain the change in GPx and GST activities in Sham rats.

Increased SOD activity was found in the Sham and CCI rats at all time points studied. SOD converts the superoxide anion to H_2_O_2_. Therefore, one would expect an increase in H_2_O_2_ levels in the spinal cord of these animals. In fact, the levels of this molecule increased in the Sham rats treated with saline for 3 and 10 days. This increase may be responsible for the increased CAT activity at day 3. CAT, an enzyme located in peroxisomes, catalyzes the breakdown of H_2_O_2_ to H_2_O and O_2_ [39]. After muscle injury, the induction of CAT is delayed and the peak of its activity occurs on the 2nd day after injury, followed by thioredoxin activity, which reaches its highest level on day 7 [40]. Thioredoxin is an antioxidant protein that limits the activity of ROS [39, 41]. With the increase in thioredoxin, CAT activity may be reduced, which explains the decrease in CAT in the saline-treated Sham rats after 10 days. Both glutathione and thioredoxin support enzyme systems for the elimination of peroxides, but each system has different kinetics and functions [41]. Thus, the probable activity of the thioredoxin does not exclude the possibility of a positive effect of the thiols found at day 10.

The presence of antioxidant molecules in the MeF may be responsible for the lack of a significant change in catalase activity in the MeF-treated Sham rats. A similar suggestion may apply to the MeF-treated CCI rats. However, catalase activity was also decreased in the saline-treated CCI rats. We suggest that this result may be related to the use of thiols, which was also decreased in these animals. The use of thiols may be related to the increase in ascorbic acid, as discussed above.

According to Goecks et al. [9], CCI injury, in contrast to the situation in Sham animals, probably mobilizes more antioxidant systems to prevent oxidative stress, given the greater excitation of the central sensory neurons. Since excessive ROS formation must be corrected only to prevent the accumulation of oxidative damage, and a slight prooxidative balance is necessary for optimal cell signaling processes [42], the different excitation of central sensory neurons may be responsible for the differences between the CCI and Sham rats. The mobilization of antioxidant systems may contribute to the absence of a significant change in lipid hydroperoxides in the spinal cord.

The lack of changes in the EPM and OF tests, plasma indicators, and body weight suggests that the MeF has no toxic effect, which raises the possibility that the MeF might be used clinically as an adjuvant to treat neuropathic pain. A recent study indicated that* S. terebinthifolius* has a toxic potential [43]. However, this study administered a treatment for 83 days, and the harmful effects occurred mainly at doses of 176 and 352 mg/kg body weight. Thus, the dose appears to be an important factor in determining the toxic effect of extracts from this plant.

## 5. Conclusion

The fractions obtained by successive extractions of dried and powdered leaves from* Schinus terebinthifolius,* particularly the MeF, contained antioxidant molecules. This is the first demonstration that MeF, in a dose of 20 mg/kg/d administered for 3 and 10 days, induces antinociception without apparent toxic effects, which suggests that this fraction could possibly be used to treat neuropathic pain. In addition, this study provided evidence that MeF administration changes prooxidant and antioxidant markers in the spinal cord, markers that had been altered after a peripheral lesion. We suggest that the changes may be related to antioxidant molecules present in the MeF, because scavenging ROS appears to present an opportunity to help to normalize the spinal-cord oxidative status altered by pain. However, we cannot exclude the possibility that other compounds of the extract may be contributing to antinociceptive effect. Further studies are necessary to better understand the relation between MeF treatment, antinociception, and oxidative stress parameters in the spinal cord of rats with CCI-induced neuropathic pain.

## Figures and Tables

**Table 1:** Antioxidant properties and chemical compounds of the fractions of *Schinus terebinthifolius*.

	*Schinus terebinthifolius *fractions				Antioxidant standard
	HeF	DcF	EtF	MeF	
Scavenging activity against hydroxyl radical in IC_50_	59.09 ± 4.8^a^	146.21 ± 6.8^b^	32.57 ± 0.8^c^	134.09 ± 7.0^b^	BHT 46.21 ± 0.8^a,c^

Scavenging activity against DPPH radical in IC_50_	596.2 ± 0.4^a^	238.99 ± 0.2^b^	5.19 ± 0.1^c^	3.00 ± 0.1^d^	BHT 6.25 ± 0.1^e^ AA 2.43 ± 0.1^f^

Total reactive antioxidant potential (TRAP)					
2.5 *μ*g/mL	1.49 ± 0.1^a^	2.32 ± 0.1^b^	1.51 ± 0.1^b^	3.22 ± 0.1^c^	ND
5.0 *μ*g/mL	1.73 ± 0.1^a^	2.39 ± 0.2^a^	2.18 ± 0.1^a^	4.02 ± 0.4^b^	ND
10.0 *μ*g/mL	1.19 ± 0.1^a^	2.42 ± 0.1^b^	3.50 ± 0.2^b,c^	4.90 ± 0.3^c^	ND
25.0 *μ*g/mL	1.27 ± 0.1^a^	2.63 ± 0.1^b^	4.265 ± 0.1^c^	5.805 ± 0.1^d^	ND

Total phenol content	7.49 ± 1.0^a^	32.27 ± 8.4^a^	357.62 ± 19^b^	406.35 ± 16.1^c^	ND

Anthraquinones	+	+	+	+	
Triterpenes/steroids	+	+	+	+	
Phenolic compounds	+	+	+	+	
Flavonoids	−	−	+	+	
Saponins	−	−	+	+	
Coumarins	−	−	−	+	
Alkaloids	−	−	−	−	

Values are expressed as mean ± S.E. Values with different superscript letters in the same row are significant different at *P* < 0.05. ND: not detected. The units are expressed as follows: TRAP (*μ*M trolox equivalents); DPPH and hydroxyl (*μ*g/mL); total phenol content (mg EGA/g fraction). +: present; −: absent.

**Table 2:** Effect of methanol fraction (MeF) from *Schinus terebinthifolius* (20 mg/kg/day) on locomotor activity and anxiety-like behaviors in naive rats.

Parameters	Elevated plus-maze test (EPM)						*P* values
	*Saline*			*MeF*			
	Mean		±SE	Mean		±SE	
% Time in open arms (s)	13.29		3.13	17.54		4.76	0.472
% Time in closed arms (s)	86.71		3.13	82.46		4.76	0.472
% Entries in open arms	21.49		3.63	19.73		4.12	0.753
% Entries in closed arms	78.51		3.63	80.27		4.12	0.753
Number of rearings in open arms	0.00		0.00	0.00		0.00	-
Number of head dips	7.42		2.36	7.42		1.82	1.0
Risk assessment (s)	16.53		4.27	19.34		3.41	0.612

Parameters	Open field test (OF)						*P* values
	*Saline*			*MeF*			
	Median	25%	75%	Median	25%	75%	

Total crossings	52.0	38.0	64.0	56.0	40.0	72.0	0.532
Peripheral crossings	45.0	35.0	50.0	44.0	36.0	50.0	0.818
Central crossings	2.0	1.0	5.0	4.0	0.0	8.0	0.447
Time in central area (s)	3.6	0.7	7.2	9.1	1.7	24.6	0.189
Latency to 1° crossing (s)	7.1	3.0	11.1	6.1	5.0	10.0	0.948
Latency to enter central area (s)	110.0	47.1	154.1	68.1	62.1	158.0	0.844
Rearings	14.0	11.0	23.0	23.0	12.0	29.0	0.292
Freezing (s)	0.0	0.0	10.8	0.0	0.0	0.0	0.108
Grooming (s)	3.9	0.0	8.1	1.0	0.0	15.0	0.946

Results are expressed as means ± SE for EPM; median and percentiles for OF, *n* = 12 rats. No significant differences were found between the groups (Student's *t*-test, for EPM; Mann–Whitney *U* test, for OF; *P* < 0.05).

**Table 3:** Effect of the methanol fraction (MeF) from *Schinus terebinthifolius *on blood parameters of liver function in naive rats.

Parameters	Experimental groups	
	Saline	MeF
AST (UI/L)	32.49 ± 1.65	27.16 ± 3.10
ALT (UI/L)	51.57 ± 1.41	47.00 ± 1.93
GGT (UI/L)	28.50 ± 3.21	24.45 ± 4.58
Total bilirubin (mg/dL)	2.07 ± 0.30	2.09 ± 0.24
Direct bilirubin (mg/dL)	1.04 ± 0.16	1.57 ± 0.31
Indirect bilirubin (mg/dL)	0.89 ± 0.19	0.52 ± 0.10

Data represent mean ± SE. *n* = 6 rats/group. AST: aspartate aminotransferase, ALT: alanine aminotransferase, and GGT: gamma-glutamyltranspeptidase.
